# Autonomic modulation: Getting it “just right”

**DOI:** 10.1016/j.hroo.2023.04.007

**Published:** 2023-05-11

**Authors:** William H. Parker, Brian Olshansky

**Affiliations:** Division of Cardiology, University of Iowa Hospitals and Clinics, Iowa City, Iowa

The autonomic nervous system (ANS) is a dense, complex, interconnected, neurohumoral network with intertwined feedback that regulates all organ systems in a plastic way under a myriad of circumstances, generally to, and for, our advantage. Occasionally, the ANS appears to misbehave or, at least, acts inexplicably counter to efficient functionality leading to heart rates that are too fast or too slow.

In this issue of *Heart Rhythm O*^*2*^, Pachon and colleagues,^1^ creative pioneers and leaders in the burgeoning field of cardiac autonomic modulation, have contributed “Cardioneuroablation: Where are We At?” They review the use of cardioneuroablation (CNA) to treat recurrent vasovagal syncope (VVS) and functional (autonomic) bradycardias without need for further therapy. In their review, Pachon and colleagues consider CNA to treat select patients with symptoms mediated by hypervagotonia. We commend their review of groundbreaking developments in ANS neuromodulation. The level of interest in CNA is growing rapidly!

Hypervagotonia, as a disease entity, though, is perplexing. High vagal tone, persistent or intermittent, associated with slower heart rates appears to be associated with good long-term prognosis.^2^^,^^3^ Episodic intense vagal discharge, by itself, is neither abnormal nor necessarily deleterious. Reasons for episodic, apparent, vagal overactivity often remain poorly understood; for the most part, no treatment is needed. If episodes of VVS are occasional, eliminating vagal connections to the heart seems overambitious, but there may be value for those with multiple, severe, recurrent unprovoked episodes of VVS or persistent symptomatic bradycardia due to high vagal activity. As no therapy is established to treat these patients, CNA is worth considering. Observational data are promising,^1^ but the data are limited and far from definitive.

Eliminating autonomic connections to halt transient overactivity may have long-lasting effects that may not be safe now or in the future. Destroying vagal inputs when the vagus is working as it should 99% of the time, but misbehaves 1% of the time, raises concern. Persistently faster heart rates with loss of vagal control, even if the heart rate is in the normal range, may ensue. Long-term consequences may emerge even if symptoms improve.^2^^,^^3^

As part of a meta-analysis that considered the use of CNA to treat VVS, Vandenberk and colleagues^4^ reported results from 11 studies (N = 337 patients) in which adverse events were evaluated after CNA. Six of these studies reported no adverse events. However, 43 (12.8%) of the 337 patients evaluated in these reports experienced adverse events including transient inappropriate sinus tachycardia and procedural complications. These adverse events represent early outcomes. It remains unclear what, if any, delayed adverse events may transpire. The exact procedure required to obtain complete benefit with little risk remains uncertain.^5^

Vandenberk and colleagues discuss dramatic benefits from CNA for VVS, mostly from small uncontrolled studies.^4^ However, a high rate of spontaneous VVS remission has been shown repeatedly in control arms of VVS studies. In one report of 51 patients with a mean of 5.5 VVS episodes during a median follow-up of 15 months, 94% remained syncope-free.^6^ However, in a meta-analysis of observational and randomized studies without CNA of patients with 2.6 ± 1.0 VVS spells per year and symptoms lasting 7.1 ± 2.6 years, 36% were syncope-free the year after diagnosis without any therapy whatsoever.^7^ If a study were to follow patients undergoing CNA vs conservative management over the long term, a similar degree of syncope remission (ie, by regression to the mean or another mechanism) may be present.^8^

Only one randomized controlled clinical trial investigated CNA to treat refractory VVS. This study showed a substantial decrease in syncope recurrence (54% in control arm and 8% in the CNA arm at 2 years).^9^ However, the study was unblinded and without a sham procedure control arm. Unfortunately, few CNA studies have proper control arms, and no CNA study has followed patients for long durations. It is known from pacemaker studies that a strong placebo (ie, sham pacemaker implantation) can apparently affect outcomes of patients with VVS. It is also known that placebo effects are prominent for therapies involving the ANS.^8^^,^^10^^,^^11^

Nevertheless, well-controlled trials have indicated that pacemakers can benefit select patients with VVS.^12^^,^^13^ Yet, no study has compared pacemakers with CNA in patients with VVS. Pachon and colleagues^14^ suggested that younger patients would be better treated with CNA than pacemakers. A study of 115 patients showed benefit with CNA in patients with VVS, including those with a vasodepressor component, suggesting that patients with VVS may benefit from CNA, irrespective of subgroup.^15^ Due to the small and largely single-center observational studies, the ideal CNA candidate has not been established. A retrospective unpublished multicenter U.S. registry was reported at the annual Heart Rhythm meeting.^16^ The data are compelling for those selected, but with a 9-month follow-up, no prospective design defining the population, and no control group, the results are inconclusive.

The recently proposed Hybrid Epicardial and Endocardial Sinus Node Sparing Ablation Therapy for Inappropriate Sinus Tachycardia (HEAL-IST) investigational device exemption (IDE) trial (NCT05280093) sets an example for a level of cautious optimism regarding trials involving autonomic modulation.^17^ In their published design study in *Heart Rhythm O*^*2*^, De Asmundis and colleagues propose a sinus node–sparing ablation approach to treat IST. We salute the efforts of De Asmundis and colleagues for providing the groundwork of their study for all to view and understand before the study is completed.

We support publishing such design studies in advance of results and welcome them in *Heart Rhythm O*^*2*^. Ideally, investigators would do well to publish their trial designs before the conclusion of the study so that all can review inclusion and exclusion criteria and better understand and comment on the study design. We further encourage a convincing, definitive, well-designed, multicenter, prospective, randomized study comparing CNA with a valid placebo that defines inclusion and exclusion criteria carefully and shows long-term benefit with no harm.

With limited trial data, we are far from a consensus regarding who should undergo CNA, what the optimal and standardized technique for CNA is, and how to follow these patients to evaluate effectiveness and harm.^5^ We hope that CNA can get the heart rate just right under all circumstances—not too fast with ablation of vagal inputs for VVS and autonomic bradycardias and not too slow with sympathetic modulation for IST, considering “The Story of the Three Bears.”^18^ When it comes to CNA to affect heart rate, we are enthusiastic, supportive, and optimistic that instead of the heart being too fast and too slow that we can get it just right. However, there is still much work to do.
